# Psoriatic transversal nail grooves on biologics

**DOI:** 10.1002/ccr3.4606

**Published:** 2021-08-25

**Authors:** Keiichi Yamanaka, Makoto Kondo

**Affiliations:** ^1^ Department of Dermatology Mie University, Graduate School of medicine Tsu Japan

**Keywords:** biologics, ixekizumab, nail, psoriasis, transverse grooves

## Abstract

We noticed transverse grooves on the nails of a psoriasis vulgaris patient being treated with ixekizumab. The indentation might have appeared during the period when the effective concentration of ixekizumab was low and psoriasis activity in the nail matrix had increased shortly before the monthly dose.

## Graphical abstract

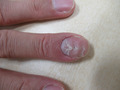

A 47‐year‐old man with a history of psoriasis vulgaris for 15 years was treated using ixekizumab, a selective humanized neutralizing monoclonal antibody against IL‐17A. The eruption was well controlled, and arthropathy did not occur. We prescribed 80 mg of Ixekizumab as a monthly self‐administered dose. We noted three transverse grooves on the patient's left middle fingernail at one consultation (Figure 1). These appeared equidistant, with normal nail gloss and quality between them. However, peripheral circulatory failure was absent. The transverse grooves that appeared similar to the annular rings of a tree were the deformed nail similar to nail dystrophy produced when the effective blood concentration of ixekizumab was low, and the psoriatic activity in the nail matrix was high, shortly before the monthly administration of ixekizumab. In contrast, the nails produced during effective antibody concentration had a normal shape. The serum ixekizumab concentration showed peak level 4 days after injection with gradual decline^1^; however, stable trough concentration during the long‐term use with individual differences.^2^ Since drug concentration was not measured in the current case, the grooves might be considered a finding indicating residual activity.

**FIGURE 1:**
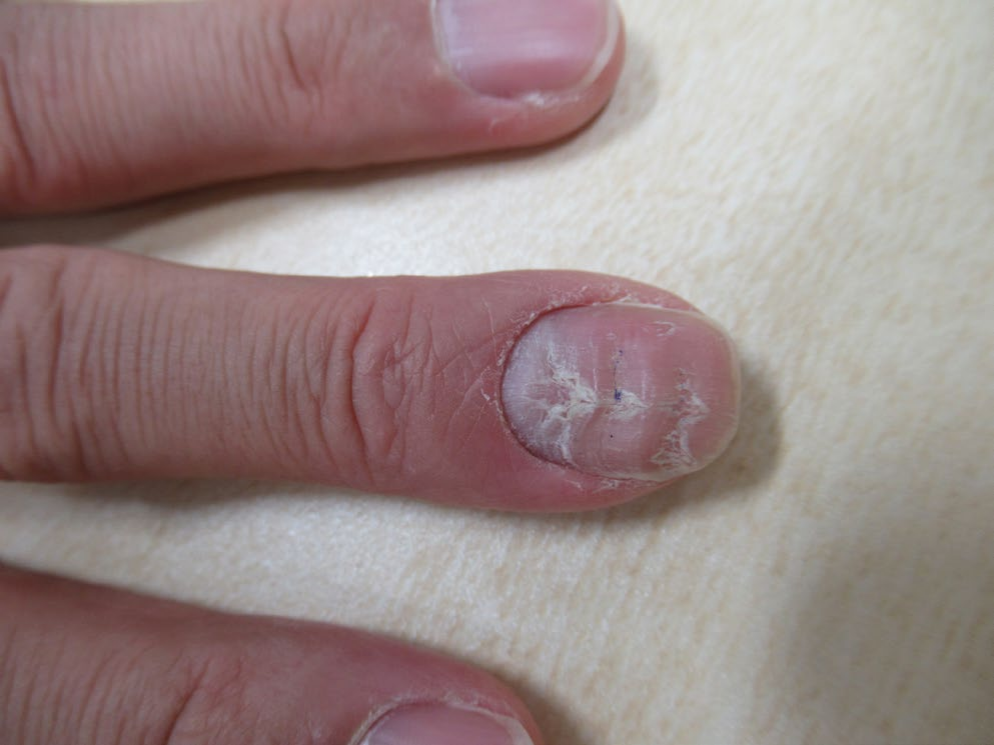
Three nearly equidistant transverse grooves with normal nail gloss and quality in between

## CONFLICT OF INTEREST

None declared.

## AUTHOR CONTRIBUTIONS

KY and MK cared for the patient, wrote the original and final manuscript. We all revised the manuscript.

## ETHICAL APPROVAL

The study was conducted in accordance with the Declaration of Helsinki. The patient provided written informed consent to publish the case, including the publication of images. The paper is exempt from ethics committee approval as only one case was reported.

## INFORMED CONSENT

Written consent for publication was obtained from the patient.

## Data Availability

The patient data are not publicly available on legal or ethical grounds.
